# Retinal Tear as a Sign of an IgA Nephropathy Flare: A Case Report

**DOI:** 10.7759/cureus.100946

**Published:** 2026-01-06

**Authors:** Ying Luu, Ray R Goshtaseb

**Affiliations:** 1 Nephrology, Ronald Reagan University of California, Los Angeles (UCLA) Medical Center, Los Angeles, USA; 2 Nephrology, University of California, Los Angeles (UCLA), Los Angeles, USA

**Keywords:** glomerular disease, iga nephropathy flare, iga nephropathy (igan), ocular manifestations, retinal tear

## Abstract

Immunoglobulin A (IgA) nephropathy is a common glomerular disease. IgA nephropathy typically presents with proteinuria, micro- or macroscopic hematuria, and/or hypertension in adults. Although rare, IgA nephropathy is also associated with ocular manifestations. Here, we present a case of retinal tear as a likely indication of IgA nephropathy flare. This case shows that it is important to assess for ocular manifestations in patients with IgA nephropathy. Ocular manifestations may assist with guiding treatment for IgA nephropathy.

## Introduction

Immunoglobulin A (IgA) nephropathy, previously known as Berger’s disease, is the most common glomerular disease worldwide [1]. The hallmark of IgA nephropathy is the predominance of IgA deposits in the glomerular mesangium, as seen on renal biopsy. Individuals with IgA nephropathy produce anti-GalNAc antibodies against a defective IgA1 molecule. This leads to the formation of IgA immune complex deposits in the kidneys, leading to a type 3 hypersensitivity reaction, causing damage to the kidneys [2]. IgA nephropathy typically presents with proteinuria, micro- or macroscopic hematuria, and/or hypertension in adults. Patients with IgA nephropathy may present with hematuria after an upper respiratory tract infection [3].

We present a case of a patient with IgA nephropathy who had been stable for many years. She was noted to have a rapid increase in proteinuria. On review of systems, a retinal tear was identified.

Although not commonly documented, upon literature review, IgA nephropathy is reported to be associated with ocular manifestations. As discussed by Oo et al., they reviewed 55 cases of ocular manifestations in patients with previously or newly diagnosed IgAN reported in 38 publications. They reported that the most common ocular manifestations of IgAN were episcleritis (23.6%), scleritis (16.4%), hypertensive retinopathy or retinal vasculopathy (20.0%), and uveitis (14.5%). The median age at presentation was 36.5 years, 54.5% of patients were female, and 61.8% had a history of IgA nephropathy before ocular involvement. Ocular manifestations were the initial presentation of IgA nephropathy in 29.1% of cases [4].

## Case presentation

A female in her 50s with past medical history of celiac disease and prediabetes has been followed for proteinuria for the past 10 years.

Table 1 summarizes her lab values. 

**Table 1:** Lab Values ^ indicates high lab values v indicates low lab values <---indicates where pt had increase in proteinuria

		Hemoglobin	Urea Nitrogen	Creatinine	Estimated GFR	HemoglobinA1C	Serum Albumin	Total Serum Protein
Ref. Range and units		11.6-15.2 g/dL	7-22 mg/dL	0.60-1.3 0mg/dL	mL/min/1.73m2	<5.7% HbA1C	3.9-5.0 g/dL	6.1-8.2 g/dL
6/25/25	7:56							
6/10/25	11:03							
6/7/25	7:41		12	0.57	>89		3.6 v	6.0 v
6/7/25	7:37							
4/28/25	12:14	14.1	18	0.58	>89			
4/13/25	7:16	12.8	17	0.6	>89	5.7 ^	3.6 v	6.2
4/13/25	7:12							
1/14/25	9:46	12.5	14	0.65	>89	5.9 ^	4.2	6.7
10/30/24	15:03	13.2						
7/7/24	7:16		16	0.68	>89	6.1 ^	4	6.9
7/7/24	7:07							
3/17/24	7:46							
3/17/24	7:35		12	0.6	>89	5.9 ^	4	6.7
12/16/23	7:24	13.1	11	0.57	>89	6.1 ^	4.4	7
12/16/23	7:18							
9/16/23	8:35							
9/16/23	7:58		15	0.67	>89	6.1 ^	4.2	6.5
6/22/23	15:40							
6/17/23	10:44							
6/17/23	9:30	14.2	11	0.61	>89	6.1 ^	4.2	7.2
3/25/23	9:04							
3/25/23	8:54		9	0.65	>89	6.1 ^	4.2	6.6
1/21/23	8:15		9	0.69	>89	6.0 ^		
1/21/23	8:05							
10/20/22	10:21		11	0.68	>89	6.1 ^	4.2	6.8
8/19/22	8:31		13	0.68	>89	5.9 ^	4.2	6.7
7/7/22	15:14							
6/29/22	8:13	12	8	0.68	>89		4.2	6.6
3/15/22	15:52						4.3	6.9
3/9/22	8:26	13.2	13	0.65			4.3	6.9
12/3/21	8:19	12.5	12	0.63			4.1	
10/19/21	7:58					6.1 ^		

Her renal function was grossly in the normal range. She had subnephrotic range proteinuria for many years. 

The patient had repeatedly refused a renal biopsy. She was treated for presumed IgA nephropathy, given her clinical history and low proteinuria. In terms of treatment, the patient was strongly advised to limit carbohydrate and sugar intake for the correction of prediabetes, which may be a cause for proteinuria. She voiced difficulty with food choices as she has celiac disease. Although angiotensin II receptor blockers (ARB), sodium-glucose cotransporter 2 (SGLT2) inhibitors, and fish oil are recommended treatments to control the progression of IgA nephropathy, she was not able to tolerate ARB, losartan, due to a complaint of paresthesia. She was unable to tolerate ARB, olmesartan, at the lowest dose, due to low blood pressure. She was able to tolerate fish oil and the SGLT2 inhibitor, dapagliflozin. 

**Figure 1:**
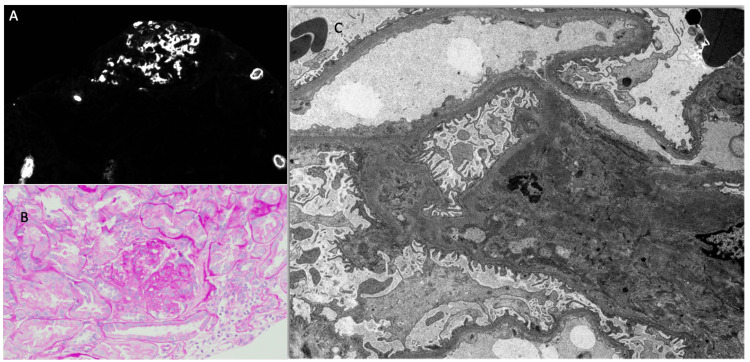
Renal biopsy. (A) Immunofluorescence microscopy was performed on frozen sections stained with fluoresceinated antisera to human IgG, IgA, IgM, C1q, C3, albumin, fibrin, and kappa and lambda immunoglobulin light chains. Staining is graded from 0 to 4+. Each frozen section consists of cortex and medulla. There are two glomeruli, one of which is globally sclerotic. The other glomerulus is on the edge but appears to have segmental sclerosis. There is mild cortical scar. Mesangial regions show strong staining for IgA (4+) as well as staining for C3 (2+) and both light chains (2+ each). No other notable staining present. (B) The specimen for conventional light microscopy, evaluated with hematoxylin and eosin stain, periodic acid-Schiff stain, periodic acid-methenamine silver (Jones) stain, and Masson's trichrome stain, consists of cortex and medulla with seven glomeruli, one of which is globally sclerotic. Two of the remaining glomeruli show segmental sclerosis with adhesion to Bowman's capsule. Mesangial regions are expanded and cellular. No endocapillary hypercellularity or crescents noted. Capillary loops are smooth. There is a mild and patchy tubulointerstitial scar. An artery shows mild intimal fibrosis. Arterioles are inconspicuous. (C) The specimen for electron microscopy, studied first by light microscopy of toluidine blue-stained one-micron-thick sections, consists of cortex with six glomeruli, three of which are globally sclerotic. The remaining glomeruli show mesangial hypercellularity with one suspicious for segmental sclerosis. No crescents noted. There is mild tubulointerstitial scar. The glomeruli are reviewed in the electron microscope. There is moderate foot process effacement. Basement membranes are segmentally wrinkled. Endothelial cells are detached in a few loops with flocculent material. No tubuloreticular inclusions are seen. Mesangial regions are expanded with cells and many immune complex deposits. The tubulointerstitial findings are confirmed.

In light of promising new treatments for IgA nephropathy, the patient later agreed to undergo a renal biopsy for definitive diagnosis. The biopsy findings were consistent with IgA nephropathy, with an Oxford classification of M1, E0, S1, T0, and C0 (Figure 1). Oxford classification to be elaborated in the Discussion section. Given the MEST score, the patient’s risk for progression of end-stage kidney disease in patients with IgA nephropathy in five years is calculated to be 4.06%. 

After the renal biopsy, she was continued on her treatment with ARB, SGLT2 inhibitor, fish oil, and diet modification. On a subsequent office visit, on review of new lab results, she was found to have developed a sudden increase in urine albumin to protein ratio from 1,800 to 3,400 in less than two months (as indicated by arrow in Table 1). This was one month after her kidney biopsy. 

On review of symptoms, the patient reported that she had a retinal tear two weeks before the lab check. She reports having been seen by an ophthalmologist at an outside clinic and was treated with laser photocoagulation. She confirmed that she did not receive corticosteroids for her retinal tear. 

At the time of the visit to ophthalmology, the patient complained of the onset of dark floaters looking like spots and lines in the right eye for two days. The patient reported occasional flashes associated with floaters, but did not see flashes for one day. The patient reported that the floaters had worsened since the onset. The patient denied any changes in visual acuity in the left eye for more than six months. She also denied the onset of flashes, floaters, wavy lines, or visual distortion in the left eye during this period. The patient declined intraocular pressure measurement.

The patient had no prior ocular history in either eye and no history of ocular surgery. She used reading glasses for vision correction.

Color fundus photography of the right eye showed retinal defects without retinal detachment but with posterior vitreous detachment. Color fundus photography of the left eye showed retinal defects without retinal detachment. The patient underwent laser retinopexy of the right eye and tolerated the procedure well, without complications.

Around the time of the retinal tear, she was preparing to move to another residence and reported eating frozen dinners due to the move. She stated that she had maintained adequate fluid intake and reported a blood pressure of 116/77 mmHg one week prior to the visit. She denied weakness, recent rashes or extremity swelling, and changes in bowel habits or weight.

Based on a review of the literature, there is evidence suggesting a link between ocular manifestations and IgA nephropathy [4]. In the setting of sudden worsening proteinuria, indicative of an IgA nephropathy flare, a steroid taper was recommended. In line with this treatment, the patient’s proteinuria improved after initiation of corticosteroid therapy.

## Discussion

IgA nephropathy occurs when IgA deposits accumulate in the kidneys, leading to local inflammation and renal injury [4]. Macroscopic hematuria occurs in 40% to 50% of patients within one to two days after an upper respiratory tract infection. Microscopic hematuria, along with mild proteinuria (less than 2 g/day), occurs in 20%-30% of patients. Nephrotic syndrome and acute renal failure occur in 5% of patients [5]. Renal biopsy provides a definite diagnosis of IgA nephropathy, where immunofluorescence staining of IgA deposits is seen with mesangial proliferation.

Patients with IgA nephropathy are evaluated for the risk of progression of renal failure. Patients with isolated hematuria, proteinuria less than 500 mg per day, and normal estimated glomerular filtration rate (eGFR) are considered at low risk for renal failure. Patients with proteinuria more than 500 mg per day, slowly decreasing GFR, and mild to moderate severity histologic findings are considered at moderate risk for renal failure. Patients with rapidly progressive proteinuria or renal biopsy with severe histologic findings are considered at high risk for renal failure [5].

In 2009, the International IgA Nephropathy Network and Renal Pathology Society developed the Oxford classification to score IgA nephropathy biopsies. The goal of this scoring system was to identify specific pathological features that predict the risk of progression of IgA nephropathy. The Oxford classification evaluates four lesions: mesangial hypercellularity (M), endocapillary hypercellularity (E), segmental sclerosis (S), and tubule-interstitial fibrosis (T). Mesangial hypercellularity (M) evaluates the number of mesangial cells per mesangial area, scored from 0 (<4) to 1 (>4). Endocapillary hypercellularity (E) evaluates an increased number of cells within glomerular capillary lumina, causing narrowing of the lumina, scored from 0 (absent) to 1 (present). Segmental sclerosis (S) measures any amount of the tuft involved in sclerosis, but not involving the whole tuft or the presence of an adhesion, scored from 0 (absent) to 1 (present). Tubule-interstitial fibrosis (T) measures the percentage of cortical area involved by the tubular atrophy or interstitial fibrosis, whichever is greater, scored as 0 (0-25%), 1(26-50%), or 2 (>50%) [6]. Crescent formations (C) were added to the Oxford classification (MEST-C) in 2017. Crescent score evaluates the presence in percent of glomeruli, scored from 0 (no crescents), 1 (crescents in <25% of glomeruli), or 2 (with crescents in >25% of glomeruli) [7]. The MEST score is used to predict the risk of end-stage renal disease in IgA nephropathy [8].

Treatment of IgA nephropathy is based on the risk of progression of kidney disease. In patients with low-risk disease progression, dietary modification, fish oil, and angiotensin-converting enzyme inhibitors (ACEi) or angiotensin receptor blockers (ARBs) are suggested to slow disease progression [5]. Recent studies also suggest the use of SGLT2 inhibitors to manage proteinuria in IgA nephropathy [9]. Patients with moderate or high risk of disease progression may be suggested to be treated more aggressively. Management for IgA nephropathy at high risk for progression includes immunosuppressive therapies such as corticosteroids, mycophenolate mofetil, rituximab, cyclophosphamide, and azathioprine in addition to treatment for IgA nephropathy with low-risk disease progression [5].

IgA vasculitis is considered a systemic form of IgA nephropathy [10]. Although rare, patients with IgA nephropathy may first present with ocular manifestations. Several case reports show the diagnosis of IgA nephropathy after presenting with ocular manifestations [11,12]. Ocular manifestations reported in patients with IgA nephropathy include uveitis, episcleritis, scleritis, retinal vasculitis, serous retinal detachment, retinal vasculopathy, and hypertensive retinopathy [4].

Bene et al. in 1984 presented a case of a 24-year-old woman with persistent microhematuria along with recurrent sinusitis and episcleritis. Kidney biopsy established a diagnosis of IgA nephropathy. Episcleral biopsy stained brightly for anti-human IgA on immunofluorescence as compared to the control biopsy, which showed no complement or immunoglobulin staining. This case report suggested a link between IgA nephropathy and ocular mucosal immunity [13].

Sakuma et al. reported a case of retinal pigment epithelial detachment associated with IgA nephropathy [14]. They suggested that in IgA nephropathy, deposition of immune complexes on the retinal pigment epithelium leads to inflammation, which may result in retinal pigment epithelial detachment.

In a systematic review, ocular manifestations of IgA nephropathy were treated with oral and topical corticosteroids, non-steroidal anti-inflammatory drugs (NSAIDs), and systemic immunosuppressants such as cyclophosphamide and azathioprine, resulting in resolution of inflammation [4].

In reviewing the patient’s timeline, her sudden increase in proteinuria was likely not due to poorly controlled blood pressure or poorly controlled diabetes. Bleeding can occur after a kidney biopsy, which could cause increased proteinuria. The patient did not report significant bleeding after the kidney biopsy, and her urine tests did not show excessive hematuria. The patient may have developed a retinal tear from moving boxes. She may also be more susceptible to a retinal tear based on her age. Although retinal tears are classically mechanical events related to vitreoretinal traction, the close temporal association between the retinal tear and worsening proteinuria raises the hypothesis that both events may have occurred during a period of increased systemic immune activity. No direct causal relationship between retinal tears and IgA nephropathy has been established.

IgA nephropathy is increasingly recognized as a systemic immune-mediated disease with documented extrarenal manifestations, including inflammatory ocular involvement. While these manifestations are typically inflammatory in nature, systemic immune activation could theoretically increase tissue vulnerability without directly causing a mechanical retinal tear.

## Conclusions

IgA nephropathy is a common glomerular disease. The typical presentation of IgA nephropathy may include mild proteinuria or hematuria following an upper respiratory tract infection. We present a case of a suspected IgA nephropathy flare occurring after many years of stable proteinuria and renal function, temporally associated with an ocular event. While we do not have histological samples of the patient’s retina at the time of retinal tear, we hypothesize that the sudden increase in proteinuria and the retinal tear occurred during the same period of systemic disease activity. However, this observation should be interpreted strictly as a temporal association and not as evidence of a causal relationship.

Although infrequent, it is important to associate IgA nephropathy with ocular manifestations. Appropriate evaluation and treatment of an IgA nephropathy flare are necessary to reduce inflammation and slow disease progression. This case highlights the importance of clinical awareness of potential ocular findings in patients with IgA nephropathy and underscores the need for multidisciplinary evaluation when systemic disease activity is suspected. Ocular findings may provide additional clinical context but should not be used in isolation to guide treatment decisions for IgA nephropathy. Further investigation is needed to determine how commonly ocular manifestations present in IgA nephropathy. Further study is also needed to understand the pathology of IgA nephropathy and its ocular manifestations.
